# Improving Physical Health Monitoring in Patients Under a London Home Treatment Team

**DOI:** 10.1192/bjo.2025.10389

**Published:** 2025-06-20

**Authors:** Inova Lee, Oluwafemi Ajose, Ana Crathorne, Brandon Wong, Rebecca Hatton

**Affiliations:** Westminster Home Treatment Team, Central and North West London NHS Foundation Trust, London, United Kingdom

## Abstract

**Aims:** Many of our patients have multiple co-morbidities in addition to their mental health diagnoses, with the two often impacting each other. Outcomes such as life expectancy in mental health patients also tend to be poorer compared with the general population. Therefore, it is vital to use the opportunity whilst patients are under our services to engage them in care for their physical health. Our quality improvement (QI) project began after reviewing home treatment team physical health policies across the UK to form a tailored local protocol, and was co-produced with our experts-by-experience. This project aimed to achieve 80% of patients under the Westminster Home Treatment Team (WHTT) with physical health monitoring completed (observations, bloods and ECGs) by Feb 2025.

**Methods:** For the 84 patients on our caseload from July–Oct 2024, data was manually collected from the electronic records for observations, blood tests and ECGs performed. Improvement strategies were implemented in 3–4 weekly cycles with input from our QI team’s nurses, doctors, support workers and our experts-by-experience in monthly QI meetings to ensure a patient-centred approach.

**Results:** Three cycles were completed with: 1) implementing a communal monitoring spreadsheet to identify patients needing checks, 2) dedicating a section for physical health in weekly MDT meetings, and 3) the formation of equipment kits for observations. The target was met for observations (50% to 90%) and bloods (20% to 81%) by the end of cycle 3, although not for ECGs at 30% to 66%, observed to likely be due to limited ECG machines available onsite. The mean time to complete observations was 7.4 days, bloods 11.2 days and ECGs 8.0 days. No patients declined observations, only 4 declined bloods and 3 declined ECGs. GPs were informed to offer checks as follow-up for any patients who did not receive them before discharge from WHTT (observations n=18, bloods n=35 and ECG n=46).

**Conclusion:** Offering physical health checks was generally received well by patients and should be integrated into routine patient contact within mental health pathways. Additional training for staff (e.g. phlebotomy), access to equipment and raising patient understanding of the physical health services available would further engagement. Ongoing collaboration between WHTT and GPs is needed for timely interventions so physical health is not neglected. Forming automated processes to capture the data collected manually will be critical for sustainability and identifying further service improvements.

